# Case report: Ultrasound-guided median nerve electrical stimulation on functional recovery of hemiplegic upper limb after stroke

**DOI:** 10.3389/fneur.2023.1244192

**Published:** 2023-11-17

**Authors:** Rui Li, Ping Zhang, Jingyi Lu, Jianlin Zhuang, Meiqi Wang, Hongmei Fang, Xiaowei Zhang, Ying Gao, Zhufen Yang, Kai Ling Chin

**Affiliations:** ^1^Department of Rehabilitation Medicine, The Second People’s Hospital of Kunming, Rehabilitation Hospital Affiliated to Kunming University, Kunming, China; ^2^Department of Biomedical Sciences, Faculty of Medicine and Health Sciences, Universiti Malaysia Sabah, Kota Kinabalu, Sabah, Malaysia; ^3^Borneo Medical and Health Research Centre (BMHRC), Faculty of Medicine and Health Sciences, Universiti Malaysia Sabah, Kota Kinabalu, Sabah, Malaysia

**Keywords:** stroke, hemiplegic upper limb, median nerve electrical stimulation, ultrasound guidance, motor function recovery

## Abstract

**Background:**

Functional restoration of hemiplegic upper limbs is a difficult area in the field of neurological rehabilitation. Electrical stimulation is one of the treatments that has shown promising advancements and functional improvements. Most of the electrical stimulations used in clinical practice are surface stimulations. In this case, we aimed to investigate the feasibility of a minimally invasive, ultrasound-guided median nerve electrical stimulation (UG-MNES) in improving the upper limb motor function and activity of a patient with right-sided hemiparesis.

**Case presentation:**

A 65-year-old male recovering from a left massive intracerebral hemorrhage after open debridement hematoma removal had impaired right limb movement, right hemianesthesia, motor aphasia, dysphagia, and complete dependence on his daily living ability. After receiving 3 months of conventional rehabilitation therapy, his cognitive, speech, and swallowing significantly improved but the Brunnstrom Motor Staging (BMS) of his right upper limb and hand was at stage I-I. UG-MNES was applied on the right upper limb for four sessions, once per week, together with conventional rehabilitation. Immediate improvement in the upper limb function was observed after the first treatment. To determine the effect of UG-MNES on long-term functional recovery, assessments were conducted a week after the second and fourth intervention sessions, and motor function recovery was observed after 4-week of rehabilitation. After completing the full rehabilitation course, his BMS was at stage V-IV, the completion time of Jebsen Hand Function Test (JHFT) was shortened, and the scores of Fugl-Meyer Assessment for upper extremity (FMA-UE) and Modified Barthel Index (MBI) were increased. Overall, the motor function of the hemiplegic upper limb had significantly improved, and the right hand was the utility hand. Electromyography (EMG) and nerve conduction velocity (NCV) tests were normal before and after treatment.

**Conclusion:**

The minimally invasive, UG-MNES could be a new alternative treatment in stroke rehabilitation for functional recovery of the upper limbs.

## Introduction

Stroke is a common global public health problem, especially in low- and middle-income countries. It is the second leading cause of death and a major cause of disability worldwide. In 2019, 12.2 million new stroke incidents were reported (1). About 70–80% of the patients have upper limb dysfunction after stroke, and 30–50% of the affected arm is still severely impaired at 6 months after a stroke. The upper extremity motor impairment significantly impedes the performance of daily activities and affects the quality of life (2). About 25–53% of stroke survivors have a dependency on at least one activity of daily living (ADL) and must often rely on family support or caregivers (3, 4). The estimated global cost of treatment, rehabilitation, and indirect costs for stroke is over US$721 billion (5).

Stroke rehabilitation mainly aims to help patients return to society and work (6). Many different physical therapies are used clinically, such as robot-assisted therapy, mirror therapy, constraint-induced movement therapy, virtual reality training, and neuromuscular electrical stimulation, among others (6–9). At present, no treatment can fully restore the function of the upper limb of hemiplegia. Electrical stimulation of the peripheral nerves can be used to activate motor and sensory fibers. Activating motor fibers or the muscles produce muscle contractions that can be used to reanimate paralyzed limbs for assistive or rehabilitative purposes (10–14). Median nerve electrical stimulation (MNES) has been used clinically as a non-invasive, surface electrical stimulation and this treatment caused changes in the cerebral blood flow pattern of the somatosensory cortex, activates multiple cortices, and induces central nerve plasticity (15). Therefore, it is widely used to promote wake-up in patients with craniocerebral trauma (16) and acute traumatic coma (17) and to enhance learning and memory (18). Also, MNES influences the functional recovery of the hemiplegic upper limb (18).

In our previous study, we applied minimally invasive ultrasound-guided median nerve electrical stimulation (UG-MNES) to treat stroke patients with relatively small cerebral hemisphere injuries and moderately impaired limbs within the best rehabilitation period of 1–3 months after the onset of the disease. The results showed the treatment had significantly improved the upper limb motor function in the immediate poststroke period (19). This paper highlights the immediate and long-term effects of the UG-MNES treatment in motor function recovery of a more severe stroke case with total upper limb disability, despite receiving the treatment 3 months after stroke, which is past the optimal window for neurological recovery.

## Case presentation

**Figure 1:**
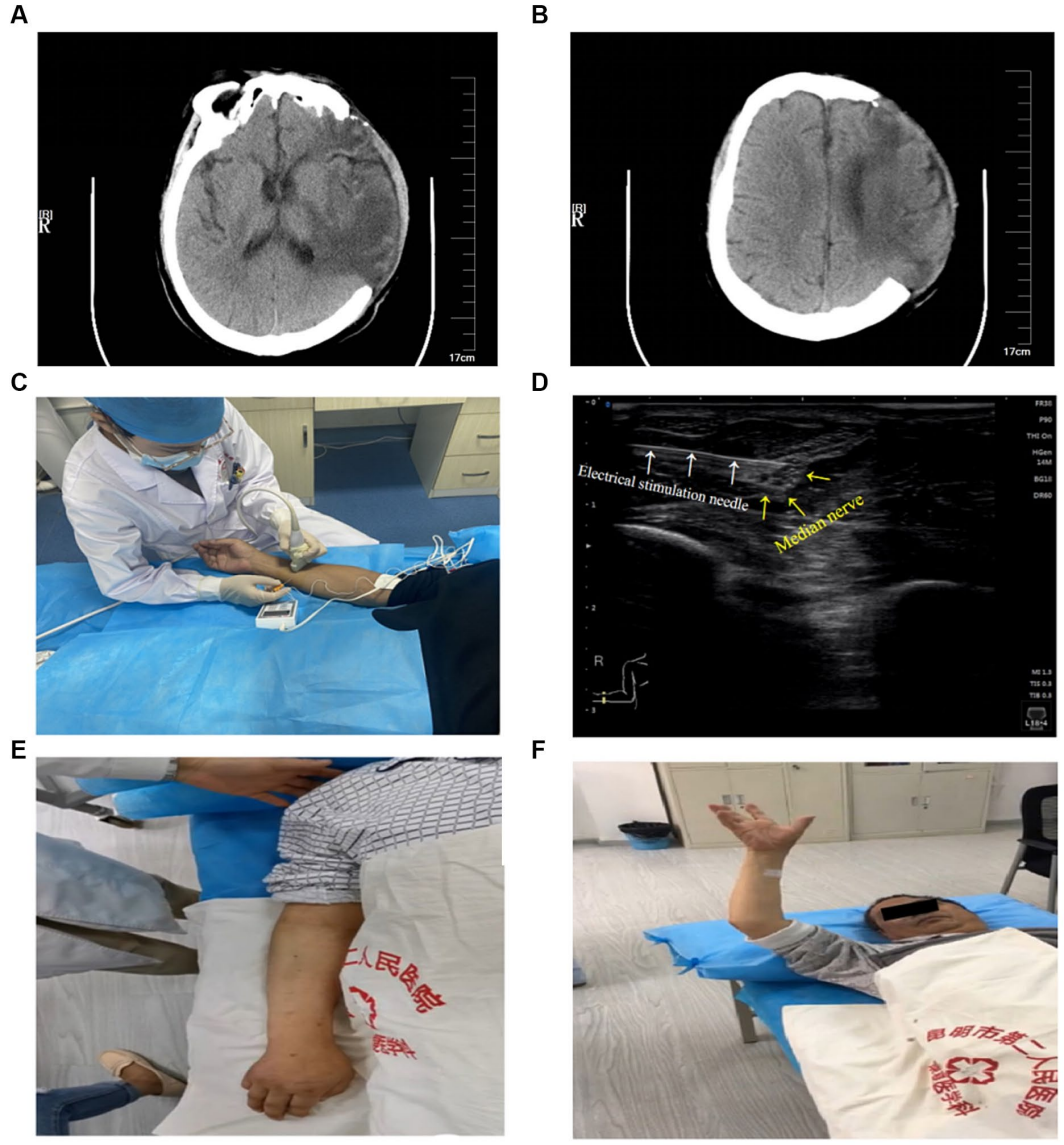
Head CT scan 2 weeks after surgery. **(A)** Dorsal thalamic level: irregular cerebral edema in the left frontal lobe, temporal lobe, and posterior limb of the internal capsule, and meningoencephalocele after the left frontotemporal parietal bone flap removal. **(B)** Lateral ventricle body level: irregular cerebral edema in left frontal and parietal lobes, and meningoencephalocele after left frontotemporal parietal bone flap removal. UG-MNES treatment and upper limb function. **(C)** Application of right median nerve electrical stimulation under ultrasound guidance. **(D)** Ultrasound image of electrical stimulation needle (white arrows) and median nerve (yellow arrows). **(E)** No casual movement of the right upper limb before the treatment. **(F)** Immediate flexion and extension of the hemiplegic upper limb and fingers after the first treatment.

A 65-year-old man had a history of hypertension for 4 years. He did not take any medication or monitor his blood pressure regularly. He was admitted to the First People’s Hospital of Kunming, China due to sudden slurred speech, nausea, and vomiting. Upon physical examination, the patient was unresponsive with the Glasgow Coma Scale (GCS) of E1 (no eye-opening), V1 (no verbal response), and M1 (no motor response) to any kind of stimuli. Bilateral pupils were dilated, and pupillary light reflex was present. Muscle strength in the extremities could not be measured. His clinical assessment with the NIH Stroke Scale (NIHSS) score was 32, implicating a severe stroke. A computed tomography (CT) scan of the head showed massive intracerebral hemorrhage in the left frontal, temporal, and parietal lobes with cerebral herniation. The diagnosis was massive cerebral hemorrhage with cerebral herniation secondary to high-risk, grade 3 hypertension. He was subjected to decompressive craniectomy and hematoma removal. After surgery, he received 2 weeks of treatments including a tracheotomy, medications for anti-infection, anti-hypertensive, and neurotrophic drugs, and hyperosmolar therapy with mannitol, and his GCS was improved to E3 (opens eyes in response to voice), VT (under tracheotomy), and M5 (move to localize pain). However, the patient experienced cognitive, speech, and swallowing disorders, irritability, agitation, behavioral disorder, urinary and bowel incontinence, and right limb immobility. The CT scan showed irregular cerebral edema in the left frontal lobe, temporal lobe, parietal lobe, and posterior limb of the internal capsule, and mild meningoencephalocele after left frontotemporal parietal bone flap removal (Figures 1A,B).

The patient was sent to the Second People’s Hospital of Kunming, China for rehabilitation treatment. He received conventional rehabilitation therapy including cognitive, swallowing, speech, physical therapy, occupational therapy, median nerve surface electrical stimulation, and traditional acupuncture therapy (total treatment time of 4 h per session, once session a day, 5 times a week). After continued active rehabilitation treatment for 3 months, the patient’s cognitive, speech, and swallowing were significantly improved. He could complete one-step instructions, give simple answers, and able to tolerate orally well. The Brunnstrom Motor Staging (BMS) of the upper limb, hand, and lower extremities of the right limb was at stage I-I-IV. The muscle strength of his right lower limb was at grade 3, and he could walk with minimal support and assistance. However, the muscle strength of his right upper limb was at grade 0 and there was no autonomous activity. The patient’s Modified Barthel Index (MBI) score was 31, Fugl-Meyer Assessment for upper extremity motor function (FMA-UE) score was 4, and he was unable to perform any of the tasks in Jebsen Hand Function Test (JHFT) (Table 1).

**Table 1:** Brunnstrom Motor Staging (BMS), Jebsen Hand Function Test (JHFT), Modified Barthel Index (MBI), and Fugl-Meyer Assessment for upper extremity (FMA-UE) before and after UG-MNES treatment.

Items	Time-point i	Time-point ii	Time-point iii	Time-point iv
BMS				
Upper limb	I	IV	III	V
Hand	I	IV	III	IV
JHFT				
Card Turning	NA	57.67	64.78	60.98
Picking up small common objects	NA	68.52	79.84	72.35
Simulated feeding	NA	88.75	98.91	92.31
Stacking checkers	NA	69.84	78.45	72.36
Moving light objects	NA	64.56	77.87	69.75
Moving heavy objects	NA	NA	99.63	86.34
FMA-UE				
Upper extremity	4	19	18	36
Wrist	0	4	0	10
Hand	0	0	0	13
Coordination/speed	0	0	0	2
*Total*	*4*	*23*	*18*	*61*
MBI				
Feeding	0	5	2	10
Chair/Bed transfer	8	8	8	12
Grooming	0	3	1	4
Toilet transfers	5	8	5	8
Bathing	0	3	1	4
Ambulation	3	3	12	15
Stairs climbing	5	5	5	8
Dressing	0	5	2	8
Bowel control	5	5	5	8
Bladder control	5	5	5	8
*Total*	*31*	*50*	*46*	*85*

Time-point – (i) before treatment, (ii) immediately after the first intervention, (iii) 1 week after the second intervention, and (iv) 1 week after the fourth intervention; NA – Completion time not available because the patient could not perform the task.

After explaining the purpose of the treatment, the procedure, and the possible risks to the patient and his family, and obtaining their consent, the patient received ultrasound-guided right median nerve electrical stimulation (UG-MNES). Upon receiving the UG-MNES treatment, the patient lay on his back, the medial side of the right forearm was fully exposed, and 7–10 cm above the Rascette lines was scanned repeatedly with short axial ultrasound to locate the median nerve. The median nerve is a circular honeycomb structure between the flexor digitorum superficialis and the flexor digitorum profundus. The median nerve of the affected upper limb was probed with a high-frequency ultrasound. The probe was adjusted until a clear image of the median nerve was observed. Electrical stimulation was delivered through a peripheral nerve stimulator (SY-708A, Su Yun, Jiangsu, China), which was pre-loaded with a disposable introducer needle (size 0.5 mm × 50 mm). The needle was inserted in-plane, avoiding the blood vessels and tendons, until the needle tip was attached to the nerve sheath as seen on ultrasound, the needle core was withdrawn, and the peripheral nerve stimulator was connected to deliver a bidirectional rectangular wave with a stimulation frequency of 2 Hz and wave width of 0.2 ms for 20 min. The stimulus current was adjusted to 1.0 mA for 5 min, followed by 1.5 mA for 15 min to trigger the thumb and forefinger palm flexion movement. This intervention was performed once a week for a total of four sessions (Figures 1C,D). During this 4-week intervention, the patient received conventional rehabilitation treatments except for median nerve surface electrical stimulation which was replaced by UG-MNES.

**Figure 2:**
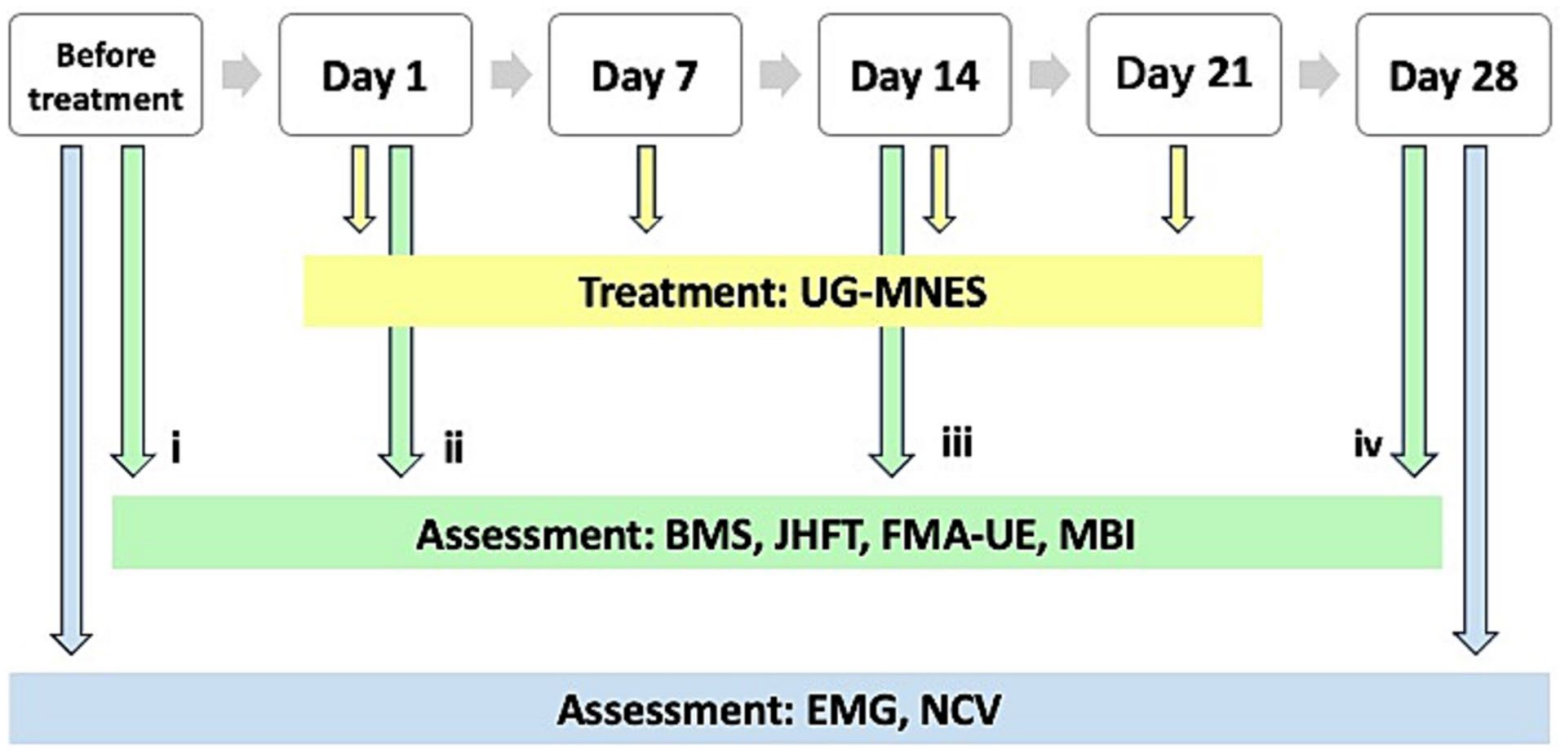
The timeline of the treatment process and assessment for the patient.

Similar to the assessment conducted before treatment (time-point i), the BMS, JHFT, FMA-UE, and MBI were used to assess the functional recovery of the right upper limb and hand after treatment at three different time-point of measurement, i.e., immediately after the first intervention to determine the effectiveness of UG-MNES treatment (time-point ii), 1 week after the second intervention (time-point iii), and 1 week after the fourth intervention (time-point iv). The third and fourth time-point measurements were used to determine the long-term functional recovery after UG-MNES treatment. Electromyography (EMG) and nerve conduction velocity (NCV) tests were performed to assess nerve damage and dysfunction before treatment and after the complete course of treatment (Figure 2).

Immediately after the first intervention, the BMS of the right upper limb and hand had improved from I-I (before treatment) to IV-IV. The patient was unable to perform any of the tasks in JHFT before treatment and regained the ability to perform all the tasks in JHFT, except moving heavy objects after the first intervention. The MBI score improved from 31 (before treatment, severe dependence) to 50, and the patient had made great progress but required assistance in feeding, toilet transfers, grooming, bathing, and dressing. The FMA score had also improved from 4 (before treatment) to 23, and immediate flexion and extension of the upper limb were observed (Table 1; Figures 1E,F).

The long-term functional recovery after UG-MNES treatment was assessed 1 week after the second and fourth interventions. The results showed that 2-week of rehabilitation was insufficient to facilitate long-term functional improvement and the motor function recovery effect was observed after 4-week of rehabilitation. After 2-week of rehabilitation, although the patient was able to perform all the tasks in JHFT, his BMS was III-III, his MBI score was 46, and his FMA score was 18. At the end of the 4 weeks of rehabilitation, his BMS was V-IV and the time to perform the tasks in JHFT was reduced. The MBI score had increased to 85 (moderate dependence) and the patient can personally perform self-feeding and ambulation, and need minimal supervision in chair/bed transfer, toilet transfers, grooming, bathing, stairs climbing, dressing, bladder control, and bowel control. The FMA score was 61, suggesting improvement in motor functioning, stability, balance, and joint functioning in the upper extremity, wrist, and hand (Table 1).

The EMG of abductor pollicis brevis (APB) muscle innervated by the median nerve showed normal spontaneous activity and interference pattern before and after UG-MNES treatment. Furthermore, based on the motor nerve conduction velocity (MNCV) and sensory nerve conduction velocity (SNCV), the onset latency and amplitude of compound muscle action potential (CMAP) and sensory nerve action potential (SNAP) before and after treatment were normal. The results of EMG and NCV indicated that UG-MNES did not cause nerve damage (Table 2).

**Table 2:** Electromyography (EMG) and nerve conduction velocity (NCV) tests before and after UG-MNES treatment.

EMG (APB)	Time-point i	Time-point iv
Lat (ms)	11.0	10.8
Amp (mV)	364	367
Multinomial wave	10.1	10.9
Early recruitment	Interference term	Interference term

NCV	MNCV		SNCV	
	Time-point i	Time-point iv	Time-point i	Time-point iv
Lat (ms)	2.86	3.65	2.44	2.31
Amp (mV)	12.5	12.4	30.1	31.5
Dist (mm)	200	195	115	115
CV (ms)	54.6	57.9	53.6	54.2
Stim (mA)	30.1	30.4	16.5	16.5

Time-point i, before treatment; Time-point iv, 1 week after the fourth intervention; EMG, electromyography; APB, abductor pollicis brevis; NCV, nerve conduction velocity; MNCV, motor nerve conduction velocity; SNCV, sensory nerve conduction velocity; Lat, latency; Amp, amplitude; Dist, distance; CV, conduction velocity; Stim, stimulation.

## Discussion

Upper limb recovery is one of the main concerns in stroke neurorehabilitation. This case demonstrated that the application of UG-MNES had improved the motor function of the upper limb and hand of a poststroke hemiplegic patient who had total upper limb disability despite receiving conventional rehabilitation during the first 3 months of the golden rehabilitation period. After 4-week of rehabilitation with UG-MNES, the patient showed good coordination and freedom of movement of all upper limb muscles, and the hand grasp abilities were restored. Although he was able to perform most of the ADLs, slightly poor distal control was observed.

The nervous system is a complicated and closed system. Peripheral stimulations such as repetitive sensory stimulation (RSS), repetitive somatosensory electrical stimulation (SES), electrical stimulation of peripheral nerves, and passive rehabilitation training can promote the plasticity of the center nervous system and improve the neural function recovery (20, 21). In this case, we applied MNES. Commonly, MNES is used as a surface electrical stimulation on comatose patients (17). The MNES could promote the regulation function of ipsilesional prefrontal areas in the functional network. Also, MNES can trigger sensorimotor stimulations of the affected hand that sequentially involve functional reorganization of distant cortical areas after stroke (18).

Physiologically, neuromuscular stimulation through electrode pads placed on the muscle belly largely activates the superficial muscles close to the skin. In order to access the deeper muscles away from the skin, larger stimulus amplitudes are required, which typically leads to more diffused recruitment of muscles, further limiting the selectivity of muscle activation (22). Therefore, the therapeutic effects of non-invasive, surface electrical stimulation are limited because there is no direct (contact) nerve stimulation, cannot be effectively positioned and applied to the nerve stem, and the adjustment of stimulus parameters is restricted (22).

The nerve fibers activated by non-invasive nerve stimulation are dependent on the location of the stimulus and the magnitude of the current (13, 22–24). Non-invasive stimulation approaches require a high compliance voltage to drive current through the high impedance of the skin to activate sufficient muscle fibers (25). Generally, the non-invasive electrical stimulation was set at a frequency of 4–50 Hz, intensity of 2–10 mA, and duration of 20–30 min/day, 5 days/week for 4–6 weeks (18, 26–31). At typically higher currents and longer pulse durations, electrical stimulation is often associated with a sharp noxious sensation, which is uncomfortable or even painful to the recipient, limiting user adoption of electrical stimulation systems. Therefore, there is a need to innovate a new electrical stimulation approach that could precisely direct the current to the underlying neural structure while activating multiple fibers using a low-voltage, comfortable current (25).

Although the non-invasive peripheral electrical nerve stimulation enhanced hand function during stroke rehabilitation through neural plasticity in the brain and the maintenance of neuromuscular junction, the transcutaneous device also stimulated some non-targeted nerves and muscles, and it is difficult to reach the deeper nerves and muscles (22). A study on invasive MNES in animal models for targeted nerve stimulation showed improved motor function of the affected forelimb in rats with hemiparesis (32). To date, only our team has reported the application of invasive MNES under ultrasound guidance to treat patients, and significant improvement in upper limb function was observed. We applied direct electrical nerve stimulation in order to target the median nerve while reducing non-targeted nerve activation. The minimally invasive UG-MNES was used at a frequency of 2 Hz by gradually increasing the stimulation intensity from 1.0 to 1.5 mA for 20 min, once a week for 4 weeks. The stimulation frequency and intensity are much lower than the non-invasive stimulation. Also, the frequency of treatment is reduced to once a week compared to traditional routine surface stimulation of once a day. This might allow outpatient patients to have high compliance and execution as well. Although this method requires direct nerve stimulation, based on the EMG and NCV test results, no presence of nerve damage was observed in this patient following the treatment.

The persistence of UG-MNES efficacy and its mechanism of action is still unclear. We considered that it might be related to the changes in central neurotransmitters after MNES, thus promoting neural network remodeling and neuron repair after stroke. This treatment contacts directly with the nerve stem, reduces the resistance and loss of surface electrical stimulation treatment conduction to the nerve stem, and achieves better motor function recovery with more comfortable electrical stimulation. Application of UG-MNES in this patient showed immediate motor function recovery despite the treatment being applied after the golden rehabilitation period, but to maintain the effectiveness, at least 4-week of rehabilitation is required.

Ultrasound imaging enabled the electrical stimulation needle to accurately contact the median nerve sheath and the current can be precisely directed to the median nerve. In this case, the minimally invasive UG-MNES is an acceptable treatment in stroke rehabilitation and has played a significant role in improving the proximal-distal upper extremity, and gross and fine functions of a hemiplegic patient in clinical application. The UG-MNES is a promising innovative electrical stimulation method in being a feasible way to generate selective upper limb motions for both assistive and rehabilitative purposes, while potentially alleviating some of the difficulties faced by other currently available electrical stimulation methods. To achieve the optimal benefits of UG-MNES in motor function recovery after stroke, we will expand the sample size and optimize parameters such as the intervention period, stimulus intensity, and interval time of stimulation in future studies.

## Data availability statement

The original contributions presented in the study are included in the article/supplementary material, further inquiries can be directed to the corresponding authors.

## Ethics statement

The study involving a human was approved by Second People’s Hospital of Kunming. The study was conducted in accordance with the local legislation and institutional requirements. The participant provided his written informed consent to participate in this study. Written informed consent was obtained from the individual for the publication of any potentially identifiable images or data included in this article.

## Author contributions

RL: study conception and design. RL, JL, ZY, MW, and XZ: data collection. RL, PZ, and JZ: analysis and interpretation of results. RL, JL, HF, and YG: draft manuscript preparation. RL and KLC: review and editing. All authors reviewed the results and approved the final version of the manuscript.
